# Arthroscopic Flexor Hallux Brevis and Plantar Capsule Release (Cochrane Procedure) for Hallux Rigidus: Case Presentation with Long-Term Follow-Up

**DOI:** 10.3390/jcm14082785

**Published:** 2025-04-17

**Authors:** Kenichiro Nakajima

**Affiliations:** Center for Foot and Ankle Surgery, Department of Orthopedic Surgery, Yashio Central General Hospital, Saitama 340-0814, Japan; nakajimakenichiro@hotmail.co.jp

**Keywords:** hallux rigidus, hallux limitus, etiology, arthroscopy, minimally invasive surgery, pathology, range of motion, cheilectomy

## Abstract

**Background**: In 1927, Cochrane observed persistent elastic resistance to hallux dorsiflexion after cheilectomy for hallux rigidus, attributing it to soft tissue tightness beneath the first metatarsophalangeal (MTP) joint. An innovative surgery was introduced using a plantar approach, dividing the plantar tissues. This procedure achieved complete pain resolution and high satisfaction in 12 patients. Despite addressing the etiology of hallux rigidus, this approach has not been adopted in current surgeries. This report presents a case treated with the arthroscopic Cochrane procedure with a long-term follow-up. **Methods**: A 73-year-old male with hallux rigidus presented with limited dorsiflexion, a painful bony prominence, and pain during walking at the first MTP joint, treated with the arthroscopic Cochrane procedure. **Results**: During surgery, hallux dorsiflexion did not improve after resecting all spurs in the MTP joint, but the dorsiflexion angle immediately improved from 55° to 85°after releasing the flexor hallucis brevis tendon, plantar capsule, and plantar portion of the lateral ligament. Improvements in both visual analog scale scores (70–0) and Japanese Society for Surgery of the Foot scores (57–88) were noted from preoperatively to 9 years and 6 months postoperatively. No postoperative cockup deformity was observed. **Conclusions**: The arthroscopic Cochrane procedure can yield favorable long-term outcomes without postoperative cockup deformity.

## 1. Introduction

Hallux rigidus (HR) is a degenerative change in the first metatarsophalangeal (MTP) joint characterized by the limited dorsiflexion of the hallux [1]. Surgical treatment is considered when conservative management is ineffective. Cheilectomy is the standard procedure for early-stage HR, whereas arthrodesis is conducted in late-stage HR [2,3,4].

In 1927, Cochrane observed that elastic resistance during hallux dorsiflexion persisted after cheilectomy, and this was attributed to the plantar soft tissues of the first MTP joint [5]. To address this, the Cochrane procedure was developed, which involves a plantar longitudinal skin incision, retracting the flexor hallucis longus muscle, and releasing the flexor hallucis brevis (FHB) tendon, plantar capsule, and plantar portion of the lateral ligament. This procedure enabled the achievement of complete pain relief and patient satisfaction in 12 patients with HR, as well as alleviating the elastic resistance during hallux dorsiflexion. This novel procedure was able to be used to identify and directly address the etiology of HR. However, no further case reports or series on the Cochrane procedure have been published since this initial report.

With the advancement of arthroscopy, it is now possible to arthroscopically approach the soft tissues released in the Cochrane procedure through the first MTP joint [6,7]. This report describes a case of HR treated with arthroscopic FHB and plantar capsule release (Cochrane procedure).

## 2. Case Presentation

### 2.1. Case Presentation

**Figure 1:**
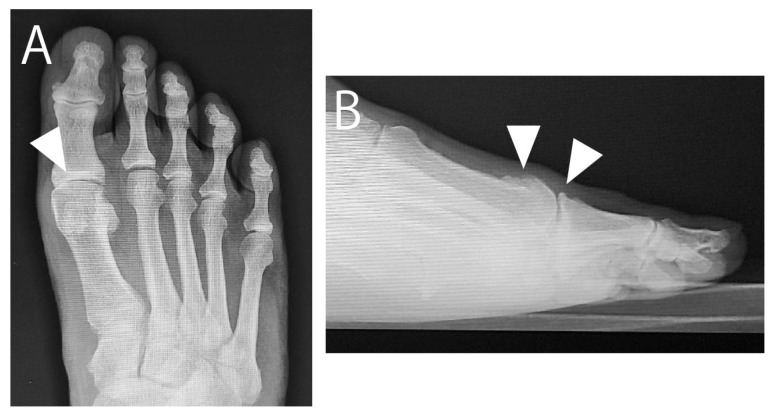
Preoperative radiographs. (**A**) Weight-bearing anteroposterior image showing narrow first metatarsophalangeal joint space (arrowhead). (**B**) Weight-bearing lateral image showing dorsal spurs on metatarsal head and proximal phalanx (arrowheads).

**Figure 2:**
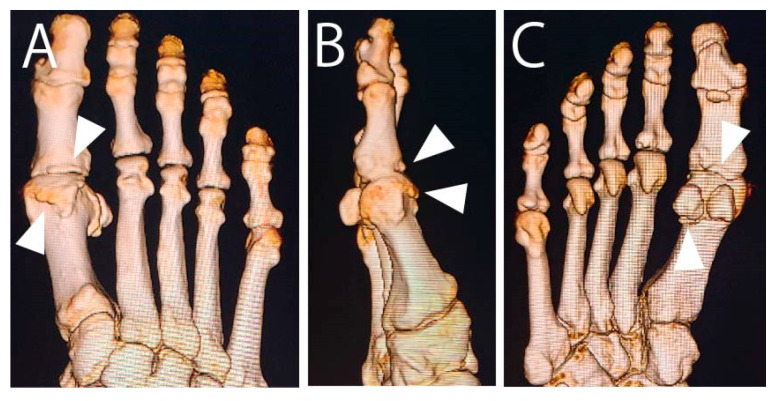
Preoperative 3D computed tomography. Spur growth is visible at distal portion of first metatarsal and proximal portion of proximal phalanx (arrowheads). (**A**) Anteroposterior image. (**B**) Lateral image. (**C**) Posteroanterior image.

In April 2015, a 73-year-old male with HR of the right foot sought consult after 1 year of unsatisfactory conservative management at a nearby clinic. He complained of a painful bony prominence, limited dorsiflexion, and pain during walking at the first MTP joint. The MTP joint was swollen, and a bony prominence at the dorsal joint was palpable. The passive dorsiflexion angle was 55°. The visual analog scale (VAS) score for pain during walking was 70, and the Japanese Society for Surgery of the Foot (JSSF) score [8,9] was 57. Radiographs showed a narrowed MTP joint space. Computed tomography revealed spur growth at the distal portion of the first metatarsal and the proximal portion of the proximal phalanx (Figure 1 and Figure 2). The patient opted for arthroscopic surgery; therefore, we proposed an arthroscopic cheilectomy, and if the improvement in dorsiflexion was insufficient, additional plantar soft tissue release could be performed.

### 2.2. Surgical Procedure

In July 2015, the patient underwent surgery. Dorsomedial, dorsolateral, proximal, and distal sesamoid portals were created [10]. Traction was applied on the hallux using a soft wire penetrating the proximal phalanx. First, all spurs shown in Figure 2 (except the spur at the plantar phalanx) were resected using a 3.0 mm hooded abrasion burr (Formula Compatible, Stryker, Kalamazoo, MI, USA) under fluoroscopic and arthroscopic guidance. However, hallux dorsiflexion did not improve enough after resecting the spurs in the MTP joint.

**Figure 3:**
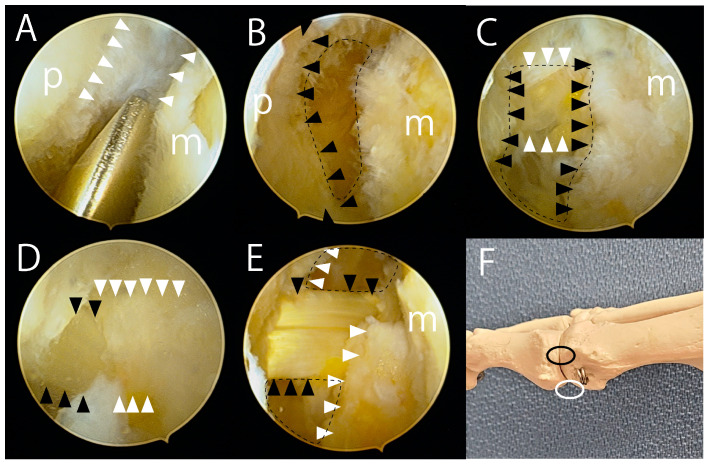
Arthroscopic views of the right foot. (**A**) The plantar capsule (white arrowheads) was visible between the base of the proximal phalanx (p) and the metatarsal head (m). The plantar capsule was resected using a cutter to expose the medial flexor hallucis brevis (FHB). (**B**) The FHB (black arrowheads) was released using a small hook-shaped knife, revealing the plantar fat pad (dotted circle). (**C**) Following the release of the FHB (black arrowheads), the sheath of the flexor hallucis longus (FHL) (white arrowheads), wrapped in fat tissue, became visible behind the residual void space (dotted circle). (**D**) The FHL sheath (white arrowheads) was debrided, exposing the FHL tendon (black arrowheads). (**E**) After releasing the lateral FHB, the void space (dotted circles) remaining after the release of the FHB tendons (white arrowheads) was visible. When the hallux was dorsiflexed, the FHL (black arrowheads) moved smoothly without restricting dorsiflexion. (**F**) A photograph showing the portals used in the arthroscopic Cochrane procedure. The black circle indicates the dorsomedial portal, and the white circle indicates the distal sesamoid portal. An arthroscope was inserted through the dorsomedial portal for visualization, and instruments were introduced via the distal sesamoid portal to release the plantar soft tissues.

Then, a 2.3 mm 30° arthroscope was inserted through the dorsomedial portal, while a 2.5 mm arthroscopic cutter (Formula Aggressive Plus; Stryker) was introduced through the distal sesamoid portal. The synovium and the capsule at the medial plantar site of the MTP joint were resected using the cutter until the medial FHB tendon became visible (Figure 3A). Subsequently, the medial FHB was released with a small hook-shaped knife (ECTRA Disposable Knife; Smith & Nephew, London, UK), exposing the plantar fat pad (Figure 3B). During FHB release, care was taken to avoid cutting the flexor hallucis longus (FHL) tendon by verifying the width of the medial sesamoid under the fluoroscopic anteroposterior view of the foot.

**Figure 4:**
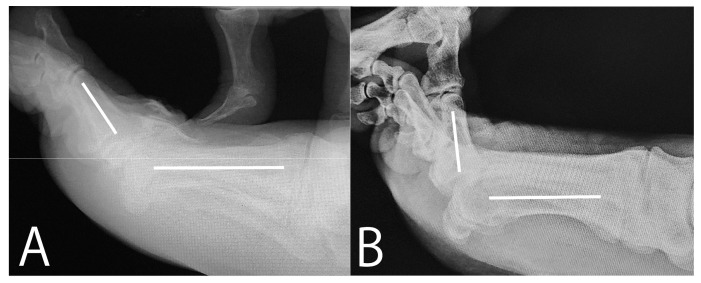
Preoperative and intraoperative radiographs. (**A**) The preoperative radiograph; the dorsiflexion angle was 55°. (**B**) The intraoperative radiograph after releasing the flexor hallucis brevis tendon and plantar capsule; the dorsiflexion angle was 85 (The white lines indicate the metatarsal and proximal phalanx axes).

After releasing the FHB, the FHL tendon sheath wrapped in fat tissue became visible behind the residual void space (Figure 3C). The sheath was debrided to reveal the FHL tendon (Figure 3D). Once the FHL tendon was identified, the lateral plantar synovium and capsule were excised to expose the lateral FHB tendon. This tendon and plantar portion of the lateral ligament were then released using a small knife. The completeness of the plantar soft tissue release, which had restricted dorsiflexion, was confirmed arthroscopically as the hallux was dorsiflexed. The FHL tendon did not impede dorsiflexion (Figure 3E,F). The dorsiflexion improved from 55° to 85° (Figure 4).

### 2.3. Postoperative Process

The patient followed up several times after surgery. However, at six months postoperatively, the patient opted to discontinue follow-up consultations since he had already achieved pain relief.

**Figure 5:**
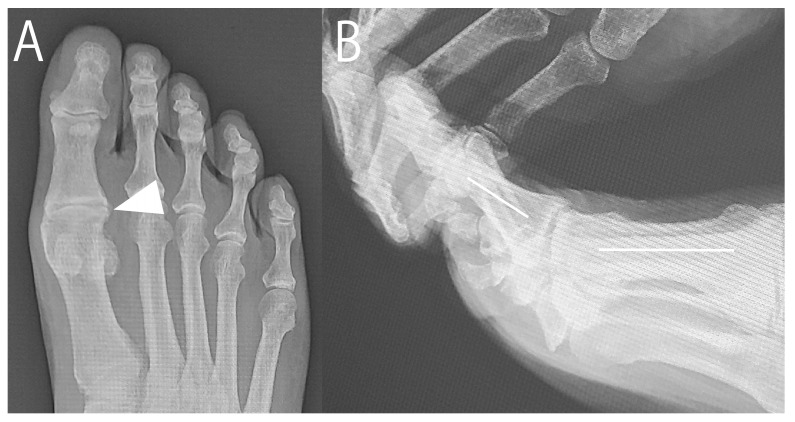
Radiographs at 9 years and 6 months postoperatively. (**A**) An anteroposterior image. Spurs at the lateral edge of the metatarsophalangeal joint were slightly enlarged compared with the preoperative radiograph (Figure 1A) (arrowhead). (**B**) A lateral image. The dorsiflexion angle was 35° (The white lines indicate the metatarsal and proximal phalanx axes). The joint space remained narrow, similar to the preoperative radiograph (Figure 1B).

**Figure 6:**
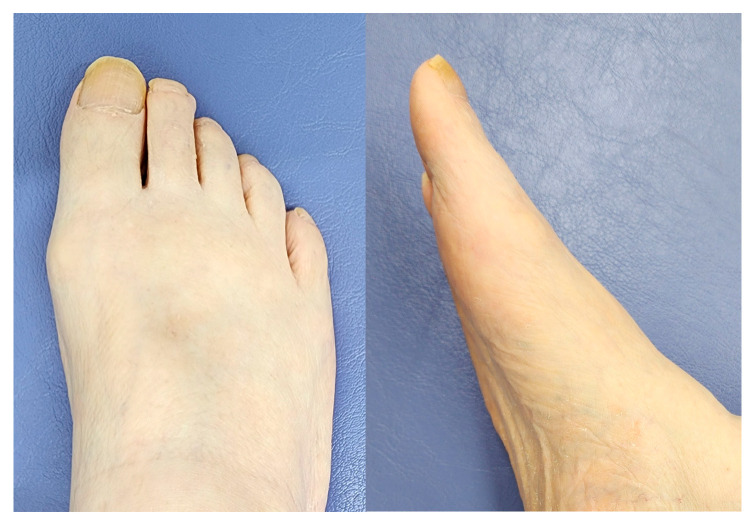
Gross appearance at 9 years and 6 months after surgery. The postoperative cockup deformity of the hallux did not occur after cutting the flexor hallucis brevis tendon.

At 9 years postoperatively, contact was re-established with the patient, who was still alive at 83 years old. He was able to follow-up with an orthopedist at a nearby clinic, whose evaluation revealed that the VAS and JSSF scores were 0 and 88, respectively. The passive dorsiflexion angle on the lateral radiograph was 35° (Figure 5). Photographs of the right foot showed no postoperative cockup deformity of the hallux after cutting the FHB (Figure 6).

## 3. Discussion

This case report presented a patient with HR who underwent arthroscopic FHB tendon and plantar capsule release (Cochrane procedure). Improvements were seen in both the VAS (70–0) and JSSF (57–88) scores from preoperatively to 9 years and 6 months postoperatively. No postoperative hallux deformity was observed.

During surgery, the dorsiflexion of the first MTP joint improved from 55° to 85° immediately after cutting the FHB tendon and plantar capsule, suggesting that the contraction of these tissues was the main cause of limited dorsiflexion in HR. Notably, the patient experienced no pain even at 9 years and 6 months postoperatively, likely because the primary cause of HR was addressed during surgery. Additionally, cutting the tendon did not cause a cockup deformity of the hallux, which is a major concern in this procedure [11]. Thus, the arthroscopic Cochrane procedure appears to demonstrate safe and effective long-term outcomes.

Cochrane identified plantar soft tissue contracture as the primary cause of HR [5]. Additionally, when the plantar tissues contract, the hallux moves like a windshield wiper around the base of the MTP joint, causing the dorsal edge of the proximal phalanx to impinge on the dorsal third of the metatarsal head [7,12]. These two key causes of HR help explain the effectiveness of current joint-preserving procedures. Cheilectomy targets dorsal impingement by removing the dorsal third of the metatarsal head [2,13]; however, it does not address plantar tissue contracture [7]. Consequently, the plantar soft tissues remain tight, maintaining pressure on the metatarsal head [7]. This limitation is reflected in the reports of poor outcomes for late-stage HR [14] and the progression of arthritic degeneration following cheilectomy [15,16]. Similarly, metatarsal head dorsiflexion osteotomy reduces stress on the dorsal surface of the metatarsal head [17]; however, it fails to address plantar tissue contracture. Poor outcomes have been reported for this procedure in high-grade HR cases [17,18], which may be attributed to the unaddressed plantar tissue contracture and continued compressive pressure on the metatarsal head [7]. In contrast, decompression metatarsal osteotomy reduces dorsal surface stress on the metatarsal head, improves dorsiflexion [19], and alleviates FHB tension by shifting the metatarsal head downward and proximally. Although this procedure does not directly release the plantar soft tissues [7], by addressing both key etiologies, studies have shown its effectiveness across all grades of HR [1,20].

We believe that the arthroscopic Cochrane procedure can be suitable for all stages of HR, considering it directly addresses plantar tissue contracture. Specifically, this procedure can be the most appropriate for patients with severe HR who present with first MTP joint ankylosis and intend to restore their range of motion because decompression metatarsal osteotomy is sufficient for treating other cases. However, studies have reported metatarsal elevation in late-stage HR [21,22]. Considerably, it remains unclear whether releasing the plantar soft tissues alone sufficiently alleviates dorsal impingement or a combination of decompression osteotomy and plantar tissue release is necessary. Further research is required to address this question.

The arthroscopic Cochrane procedure is a technically demanding yet safe procedure when performed correctly. The initial step involves the fluoroscopic resection of spurs. As the spurs occupy the joint space, the capsule is tight, and the joint space is narrow. Removing the spurs slightly widens the joint space, facilitating subsequent arthroscopy. However, the fluoroscopic removal of spurs at the plantar phalanx and distal sesamoid is not recommended, as it may damage the FHL tendon. In the secondary step, the arthroscopy of the plantar aspect of the first MTP joint in HR is challenging because of the abundance of synovium and the continuity of structures, including the FHB sheath, the capsule, the periosteum of the distal sesamoid spur, and the cartilage of the sesamoid—all of which appear uniformly white. Therefore, we consider verifying the position of the sesamoid under fluoroscopic guidance and resecting the plantar soft tissue using a small arthroscopy cutter to be the most reliable approach. Releasing the medial FHB tendon further widens the joint space, making it easier to identify the FHL tendon sheath. The small cutter remains a safe tool for subsequently identifying the FHL tendon. Once the FHL tendon is identified and secured, releasing the lateral FHB tendon becomes straightforward. Lastly, the plantar soft tissues are arthroscopically examined to ensure they do not restrict hallux dorsiflexion. If the plantar portion of the lateral ligament is not completely released, dorsiflexion may remain limited.

This case report has several limitations. First, because this procedure was only performed in one case, the results and conclusions cannot be generalized. Further research, such as case series treated with this procedure, comparative studies, and meta-analyses, is necessary for evaluating its effectiveness. Second, because of the 9-year gap between the last two consultations, it was unclear whether the decrease in hallux dorsiflexion was caused by the effects of surgery or degenerative change. Third, the condition of the released FHB could not be evaluated since the final consultation was conducted by a different orthopedist. Finally, this procedure is technically demanding. Therefore, using alternative surgical settings or having limited experience with first MTP joint arthroscopy may lead to unexpected difficulties.

## 4. Conclusions

This case demonstrates that the arthroscopic Cochrane procedure can achieve favorable long-term outcomes without postoperative cockup deformity, even at 9 years and 6 months postoperatively.

## Data Availability

The data for the present case are available from the author on reasonable request.

## Abbreviations

The following abbreviations are used in this manuscript:

HR	Hallux rigidus
MTP	Metatarsophalangeal
FHB	Flexor hallucis brevis
FHL	Flexor hallucis longus
VAS	Visual analog scale
JSSF	Japanese Society for Surgery of the Foot
